# Editorial: Case reports in pediatric surgery 2022

**DOI:** 10.3389/fped.2023.1179772

**Published:** 2023-05-02

**Authors:** Gloria Pelizzo, Luca Pio, Jürgen Schleef

**Affiliations:** ^1^ Department of Pediatric Surgery, “V. Buzzi” Children's Hospital and University of Milan, Milan, Italy; ^2^ Department of Surgery, St. Jude Children's Research Hospital, Memphis, TN, United States; ^3^ Department of Pediatric Surgery and Urology, Institute for Maternal and Child Health—IRCCS “Burlo Garofolo”, Trieste, Italy

**Keywords:** pediatric case report, pediatric surgery, pediatrics, surgical reports, pediatric surgery education

**Editorial on the Research Topic** Case reports in pediatric surgery 2022

Pediatric surgery is one of the youngest surgical specialties, covering a broad spectrum of acquired pathological conditions and congenital malformations. In addition, the particularity of covering different surgical subspecialties, such as thoracic, digestive, urologic, and oncologic, makes pediatric surgery one of the richest medical fields in terms of case reports.

## Congenital diseases

Neonates could be affected by a large spectrum of malformations with different incidences, and thus, pediatric surgeons are faced with the challenge of managing unique and difficult-to-handle cases during their practice. In “Ectopic scrotum and penoscrotal transposition: Case report and literature review,” Huang reports a rare association of genital malformations and VATER/VACTERL syndrome, providing a technical pictorial description of a combined laparoscopy exploration, rotation flap scrotoplasty, and orchiopexy.

In “Congenital perineal lipoma associated with additional external genitalia anomalies”, Tocchioni et al. reports about an additional association of genital malformation and benign tumor in newborns, discussing pathogenesis, preoperative assessment, and surgical management.

In “Meconium peritonitis, intestinal atresia combined with biliary atresia: A case report”, Han presents a complex association of multiple digestive malformations such as intestinal and biliary atresia with m peritonitis, describing emergency surgical management and the following biliary surgery.

Despite inguinal hernia management being extensively described in the literature, some unusual presentations, as described by Nakagawa in “A giant bilateral inguinal hernia requiring artificial mesh and multi-stage surgery in infancy; hernioplasty with silo placement to prevent acute compartment syndrome”, were not included in the international guidelines, which necessitated a tailored approach (1).

Tracheoesophageal fistula recurrence is another neonatal controversial condition, and despite several described techniques, little is known regarding the best management of this postoperative complication (2). In “Refractory tracheoesophageal fistula treated using multistage surgery: A case report”, Nakagawa et al. reports and discusses several techniques and the effectiveness of subtotal esophageal resection with gastric tubulization. Endocrine surgery is rarely reported in children. In “Pylorus-preserving pancreatoduodenectomy for focal congenital hyperinsulinism in a 5-month-old baby”, Spagnoletti reports about an innovative technique of pancreatoduodenectomy with pyloric preservation, while Calcaterra et al. describes a rare case of hyperparathyroidism in “Hypercalcemia and neurological symptoms: A rare presentation of hyperfunctioning parathyroid adenoma in an adolescent.”

Congenital malformations can be diagnosed later in childhood, as described by Xiong in “A case of giant accessory hepatic lobe torsion combined with left hepatic vein branch thrombosis in a child,” with a rare hepatic malformation requiring a laparoscopic resection of an accessory hepatic lobe.

## Acquired diseases

Apart from elective and emergency procedures, pediatric surgery includes the management of all complications of children’s clinical management from birth to adolescence. In “A report on the countermeasures after the rupture of the scalp venous indwelling needle catheter in 12 cases”, Zhong discusses the imaging and surgical management of a needle catheter iatrogenic complication in children.

Pediatric surgeons usually handle challenging cases of patients requiring a structured differential diagnosis, as described by Jiang in “Ultrasound-guided percutaneous drainage of iliopsoas abscess with septicemia in an adolescent: A case report and literature review,” warranting an early diagnosis of a rare abscess in a kid with general symptoms and signs.

Traumatic injuries are the leading cause of pediatric mortality; however, a majority of patients can benefit from nonsurgical management (3). Liu et al. confirms the relevance of a non-surgical approach, highlighting the effectiveness of interventional radiology in “Severe blunt liver injury complicated by delayed massive hemobilia in a toddler: A case report and literature review.”

Wang points out the relevance of the non-surgical approach in “Hysteroscopy combined with a vaginal mold for severe recurrent vaginal adhesion and stenosis with pyocolpos after pelvic fracture in a 13-year-old female,” describing an endoscopic technique for long-term sequelae of pelvic fractures.

## Oncology diseases

Over the years, pediatric surgical oncology has acquired increased relevance in children's survivorship, with an active role in all steps of oncology management, including chemotherapy sequelae (4). Zarfati describes an unusual chemotherapy-induced lung cavitation and its surgical management in “Chemotherapy-induced cavitating Wilms’ tumor pulmonary metastasis: Active disease or scarring? A case report and literature review.” Oncology care, including chemotherapy administered by using a central venous catheter, can lead to potential surgical management in case of complications, as described by D'Angelo et al. in “Case report: Bilateral pleural effusion secondary to late migration of a tunneled central venous catheter in a patient affected by high-risk neuroblastoma.”

Digestive surgery in neonates could lead to a challenging and life-threatening situation (5). In “Case report: Spindle cell neoplasm presenting as a spontaneous intestinal perforation in a term infant”, Callaghan describes a rare case of neonatal intestinal perforation related to a spindle cell tumor, focusing on genetic assessment and the molecular signature of this particular neoplasm.

Newborns may present with embryonic tumors detected by the prenatal ultrasound, as described by Liu in “Fetal mesenchymal hamartoma of the liver: A case report and literature review,” with an additional extensive systematic review.

Some secondary tumors can occur during chemotherapy treatment. In “Experience of a rare case of rebound of the Kasabach-Merritt phenomenon during sirolimus treatment in kaposiform hemangioendothelioma”, Wang describes a successful interventional radiology management of a secondary vascular tumor caused by consumptive coagulopathy for treating thrombocytopenia induced by chemotherapy.

Surgical oncology usually requires demolitive procedures to obtain macroscopic gross resection with tumor removal. However, some clinical presentations require minimal surgery as discussed by Persano in “Primary ovarian Burkitt's lymphoma: A puzzling scenario in the pediatric population.” The need for adopting conservative approaches in some pediatric surgical oncology scenarios is also stressed by Romeo in “Minimally invasive management of a giant para tubal cyst in an adolescent female: Case report and review of the literature in the pediatric population,” highlighting the role of ovarian-sparing surgery for fertility preservation.

The increase in survival rates in children with cancer led to increased studies of long-term functional sequelae; therefore, fertility preservation became a relevant topic in pediatric surgical oncology (6). Martucci et al. describes the surgical resection of a massive uterine leiomyoma with fallopian tube preservation in “Uterine leiomyoma in pediatric population: A case report and review of the literature.”

In this research topic of Frontiers in Pediatrics, we have selected different case reports involving unexpected/unusual diagnoses, decision-making, and clinical management.

This research topic and all the aforementioned case reports describe the variability of pediatric surgical disorders and the ways and means to treat them and to manage rare disease conditions.

## References

[1] Morini F Dreuning KMA Janssen Lok MJH Wester T Derikx JPM Friedmacher F Surgical management of pediatric inguinal hernia: a systematic review and guideline from the European Pediatric surgeons’ Association Evidence and Guideline Committee. Eur J Pediatr Surg. (2022) 32(3):219–32. 10.1055/s-0040-1721420 33567466

[2] Lelonge Y Varlet F Varela P Saitúa F Fourcade L Gutierrez R Chemocauterization with trichloroacetic acid in congenital and recurrent tracheoesophageal fistula: a minimally invasive treatment. Surg Endosc. (2016) 30(4):1662–6. 10.1007/s00464-015-4352-1 26139499

[3] Leung-Tack M Ong EGP McGuirk S. Interventional radiology and open surgery: an effective partnership for solid organ trauma. J Pediatr Surg. (2022) 57(2):266–70. 10.1016/j.jpedsurg.2021.10.037 34838307

[4] von Allmen D. Pediatric surgical oncology: a brief overview of where we have been and the challenges we face. Semin Pediatr Surg. (2019) 28(6):150864. 10.1016/j.sempedsurg.2019.150864 31931962

[5] Federici S De Biagi L Straziuso S Leva E Brisighelli G Mattioli G Multicenter retrospective study on management and outcome of newborns affected by surgical necrotizing enterocolitis. Minerva Chir. (2017) 72(3):183–7. 10.23736/S0026-4733.17.07159-0 28150915

[6] Mulder RL Font-Gonzalez A Hudson MM van Santen HM Loeffen EAH Burns KC Fertility preservation for female patients with childhood, adolescent, and young adult cancer: recommendations from the PanCareLIFE consortium and the international late effects of childhood cancer guideline harmonization group. Lancet Oncol. (2021) 22(2):e45–56. 10.1016/S1470-2045(20)30594-5 33539753

